# Short-term comparison between navigated subthreshold microsecond pulse laser and oral eplerenone for chronic central serous chorioretinopathy

**DOI:** 10.1038/s41598-022-08764-2

**Published:** 2022-03-18

**Authors:** Lisa Toto, Rossella D’Aloisio, Chiara De Nicola, Federica Evangelista, Maria Ludovica Ruggeri, Luca Cerino, Maria Beatrice Simonelli, Agbéanda Aharrh-Gnama, Marta Di Nicola, Annamaria Porreca, Rodolfo Mastropasqua

**Affiliations:** 1 Ophthalmology Clinic, Department of Medicine and Science of Ageing, University G. D’Annunzio Chieti-Pescara, Chieti, Via dei Vestini 31, 66100 Chieti, Italy; 2 Laboratory of Biostatistics, Department of Medical, Oral and Biotechnological Sciences, University “G. d’Annunzio” Chieti-Pescara, Chieti, Via dei Vestini 31, 66100 Chieti, Italy; 3 Institute of Ophthalmology, University of Modena and Reggio Emilia, Modena, Italy

**Keywords:** Diseases, Eye diseases, Retinal diseases, Outcomes research

## Abstract

To compare the anatomical/functional changes after navigated subthreshold pulse laser (SML) and oral eplerenone therapy for chronic central serous chorioretinopathy (cCSC). A total of 36 eyes of 36 patients suffering from cCSC treated with navigated SML (Navilas® 577s; OD-OS GmbH, near Berlin, Germany) (18 eyes, SML group) and oral eplerenone (18 eyes, eplerenone group) were enrolled in this retrospective study. Main outcome measures during a 3-month follow up period included changes of best corrected visual acuity (BCVA), central macular thickness (CMT), foveal subretinal fluid thickness (FSRFT), and subfoveal choroidal thickness (SFCT). At baseline average duration of symptoms was 6.8 ± 0.6 months in SML group and 6.4 ± 0.9 months in eplerenone group (*p* = 0.127). Mean BCVA, CMT and FSRFT changed significantly over time (*p* < 0.001). From baseline to 90 days the BCVA improved from 0.3 ± 0.1 to 0.1 ± 0.1 logMAR in SML group and from 0.3 ± 0. to 0.2 ± 0.1 logMAR in eplerenone group, CMT reduced from 357.1 ± 104.3 to 210.6 ± 46.7 μm and from 428.7 ± 107.7 to 332.5 ± 27.5 μm in SML group and eplerenone group respectively, FSRFT reduced from 144.4 ± 108.2 to 22.6 ± 37.2 μm and from 217.1 ± 105.9 to 54.4 ± 86.2 μm in SML group and eplerenone group. 55.6% of patients in SML group and 66.7% in eplerenone group showed a complete resolution of FSRFT during follow up. The interaction between group and time was statistically significant with greater absolute variation for CMT and FSRFT in SML group compared to eplerenone group (*p* < 0.001 and *p* = 0.043). SFCT did not change significantly during follow up (*p* = 0.083) for both groups. Both navigated SML and oral eplerenone were effective treatments showing recovery of retinal morphology and related visual acuity improvement in cCSC.

## Introduction

Central serous chorioretinopathy (CSC) is an important macular disease characterized by serous detachment of the neurosensory retina with or without pigment epithelium detachment (PED), with a male prevalence^1^.

It is a pachychoroid-spectrum disease characterized by dilated and hyperpermeable choroidal vessels with increased hydrostatic pressure, causing focal Bruch's membrane and retinal pigment epithelium (RPE) disruption leading to a serous retinal detachment^2^.

Usually acute CSC resolves spontaneously within months, although its recurrence has been reported in 30.0–50.0% of patients within the first year from the first episode, whilst 5–10.0% develop chronic CSC (cCSC) characterized by persistent subretinal fluid (SRF) for longer than 4–6 months^3–5^. Chronicization is characterized by neurosensory retinal atrophic changes and widespread RPE alterations with irreversible photoreceptor damage and permanent visual loss. In addition, choroidal neovascularization (CNV) and cystoid macular degeneration are possible complications^6^. Laser photocoagulation treatment and photodynamic therapy (PDT) are possible treatment options for CSC^7,8^ nevertheless several possible complications have been described^8–10^. The efficacy of mineralcorticoids receptor (MR) inhibitors therapy reported in several studies has recently become controversial after a study comparing eplerenone to placebo has shown its not-superiority and the authors have suggested to discontinue the prescription^11^.

Subthreshold microsecond pulse laser (SML) has been proposed as an effective method to treat cCSC^6,12–18^.

SML consists of an application of short duration subthreshold laser spots stimulating the RPE, this results in the production of so called “heat shock proteins”, which act as chaperone proteins, aid in refolding denatured proteins and are protective against apoptosis and inflammation. By normalizing RPE function, SML improves the transretinal pump to reabsorb subretinal fluid^19–21^.

Recently non-contact navigated devices improved the efficacy and safety of the SML procedure in several retinal disorders including cCSC^22–24^. Navigated laser therapy uses an eye-tracking laser delivery system with the possibility to overlay retinal images on to the real time-fundus image. Registered image overlays allow the surgeon to map and target precise treatment areas while the eye-tracking system compensates for patient movement. In addition, preset grid patterns with equidistant spacing or confluent spots can be delivered semi-automatically to the planned treatment area with precision increasing the accuracy of laser delivery.

The aim of this study was to compare retrospectively anatomical and functional changes of cCSC eyes treated with a single session of navigated 577-nm yellow SML and oral eplerenone in a 3-month follow up study.

## Results

We evaluated 36 eyes of 36 patients (30 males and 6 females) with cCSC, with a mean age of 48.9 ± 8.6 years in SML group and 52.2 ± 7.9 in oral eplerenone group (*p* = 0.409). Average duration of symptoms with fundus and optical coherence tomography (OCT) evidence of SRF persistence was 6.8 ± 0.6 months (range from 6 to 8 months) in SML group and 6.4 ± 0.9 months (range from 6 to 8 months) in eplerenone group (*p* = 0.127). The overall features of all eyes at baseline are summarized in Table 1. All diseased eyes (36) showed foveal SRF and cCSC was a primary disease in 77.8% of SML group eyes and 88.9% of eplerenone group eyes. In 8 eyes (44.4%) of the SML group and 6 eyes (33.3%) of the eplerenone group there was widespread RPE atrophy not involving the fovea in any of the cases. In 12 eyes (66.7%) of the SML group and 13 eyes (72.2%) of the eplerenone group there was a PED. In 1 eye of the eplerenone group there was intraretinal fluid.

**Table 1:** Baseline clinical characteristics of patients.

Ocular characteristics	SML group (n = 18)	Eplerenone group (n = 18)	*p* value
Gender, n (%)			0.655
Males	14 (77.8)	16 (88.9)	
Females	4 (22.2)	2 (11.1)	
Baseline visual acuity (logMAR), mean ± SD	0.3 ± 0.1	0.3 ± 0.1	0.720
SE (diopters)	− 0.4 ± 0.4	− 0.5 ± 0.4	0.458
CMT (µm), mean ± SD	357.1 ± 104.3	428.7 ± 107.7	0.051
FSRFT (µm), mean ± SD	144.4 ± 108.2	217.1 ± 105.9	0.051
SFCT (µm), mean ± SD	383.2 ± 70.0	452.9 ± 127.9	0.053
Primary disease, n (%)	14/18 (77.8)	16/18 (88.9)	0.655
Foveal SRF, n (%)	18/18 (100.0)	18/18 (100.0)	1.000
Widespread RPE atrophy, n (%)	6/18 (33.3)	8/18 (44.4)	0.733
Subtle RPE changes/focal RPE atrophy, n (%)	12/18 (66.7)	10/18 (55.6)	0.732
PED, n (%)	12/18 (66.7)	13/18 (72.2)	0.815
Intraretinal fluid, n (%)	0/18 (0.0)	1/18 (5.6)	0.999
CH (%)	17/18 (94.4)	16/18 (88.9)	1.000

BCVA, best-corrected visual acuity; LogMAR, logarithm of the minimum angle of resolution; SE, spherical equivalent; FSRFT, foveal subretinal fluid thickness; CMT, central macular thickness; SFCT, subfoveal choroidal thickness; foveal SRF, foveal subretinal fluid; RPE retinal pigment epithelium; PED, pigment epithelium detachment; CH, choroidal hypermeability.

No active CNV was detected in any of the diseased and fellow eye.

At baseline there is marginal significance in CMT, FSRFT, and SFCT in terms of best corrected visual acuity (BCVA), subfoveal choroidal thickness (SFCT), central macular thickness (CMT) and FSRFT between groups (Table 1).

BCVA, CMT and foveal subretinal fluid thickness (FSRFT) changed significantly during follow-up period in both groups (*p* < 0.001) (Table 2). From baseline to 90 days the BCVA improved from 0.3 ± 0.10 to 0.1 ± 0.1 logMAR in SML group and from 0.3 ± 0.1 to 0.2 ± 0.1 logMAR in eplerenone group, CMT reduced from 357.1 ± 104.3 to 210.6 ± 46.7 μm and from 428.7 ± 107.7 to 332.5 ± 27.5 μm in SML group and eplerenone group respectively, FSRFT reduced from 144.4 ± 108.2 to 22.6 ± 37.2 μm and from 217.1 ± 105.9 to 54.4 ± 86.2 μm in SML group and eplerenone group. The interaction between group and time was statistically significant with greater variation for CMT and FSRFT in SML group compared to eplerenone group (*p* < 0.001 and *p* = 0.043) (Table 2).

**Table 2:** Change over time of BCVA (best corrected visual acuity), CMT (central macular thickness), FSRFT (foveal subretinal fluid thickness) and SFCT (sub foveal choroidal thickness) values in the SML group and the eplerenone group.

Variable	Group	Baseline	30 days	60 days	90 days	*p* value		
						Group^a^	Time^b^	Interaction^c^
BCVA (logMAR)	SML	0.3 ± 0.1	0.1 ± 0.1	0.1 ± 0.1	0.1 ± 0.1	0.887	< 0.001	0.353
	Eplerenone	0.3 ± 0.1	0.1 ± 0.1	0.1 ± 0.1	0.2 ± 0.1			
CMT (μm)	SML	357.1 ± 104.3	250.2 ± 57.2	217.0 ± 31.3	210.6 ± 46.7	0.002	< 0.001	< 0.001
	Eplerenone	428.7 ± 107.7	289.6 ± 77.3	267.7 ± 85.8	332.5 ± 27.5			
FSRFT (μm)	SML	144.4 ± 108.2	56.3 ± 42.8	32.8 ± 26.4	22.6 ± 37.2	0.042	< 0.001	0.043
	Eplerenone	217.1 ± 105.9	219.4 ± 108.9	95.0 ± 63.5	54.4 ± 86.2			
SFCT (μm)	SML	383.2 ± 70.0	376.1 ± 45.4	354.4 ± 37.7	343.8 ± 49.1	0.184	0.083	0.071
	Eplerenone	452.9 ± 127.9	386.9 ± 125.9	354.9 ± 94.3	387.4 ± 130.3			

Data are expressed as mean and standard deviation. Probability that effects of group on evaluated variables is influenced by:

^a^Groups, for each variable, the differences has been tested between groups over times.

^b^Time, for each variable, the differences have been tested between baseline, 30, 60 and 90 days of the two groups.

^c^Probability that the effect of nutritional intervention is greater in one distinct group (interaction surgery*group).

SML: subthreshold pulse laser.

## SFCT did not change significantly during follow up in each group (*p* = 0.083) and did not show differences between the two groups (*p* = 0.184)

**Figure 1:**
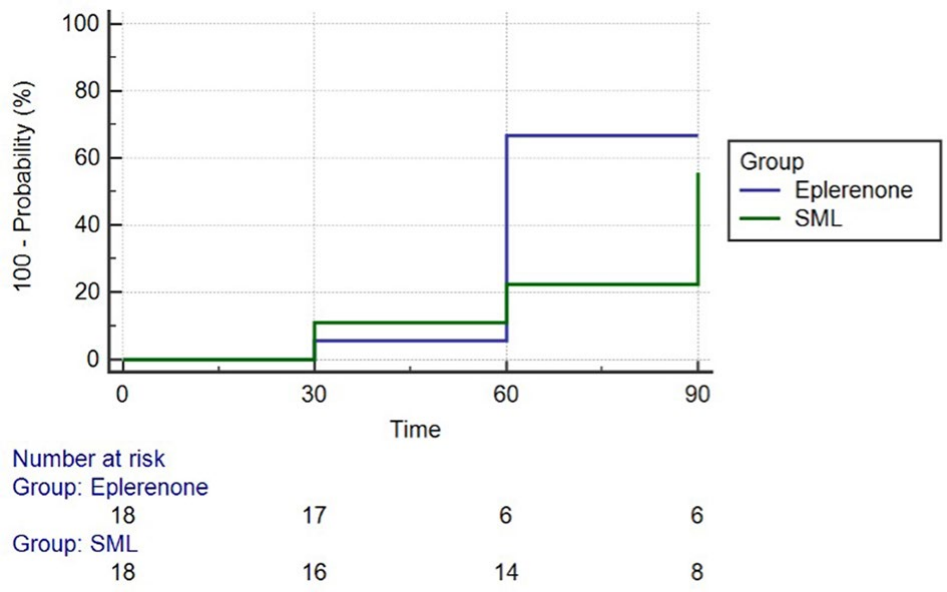
Probability of recovery and patients at risk at each considered time point in SML group and eplerenone group. The horizontal axis (Time) represents time in days, and the vertical axis shows the probability of recovery. The complement of Kaplan–Meier curve shows a vertical drop in presence of an event.

Figure 1 shows the probability of resolution of foveal SRF in SML group and eplerenone group. In SML group at 30 days the 2 out of 18 patients showed resolution of foveal SRF, at 60 days 22.2% of patients and at 90 days 55.6% of patients (Fig. 1). In eplerenone group at 30 days 1 out of 18 patients showed a resolution of FSFR and at 60 and 90 days 66.7% of patients showed foveal SRF resolution. The percentage of patient recovery was statistically different between the two groups at 60 days (*p* = 0.019). Planned contrasts’ on t-statistic confirm mean differences between baseline and 90 days for BCVA, CMT and FSRFT in each group.

**Figure 2:**
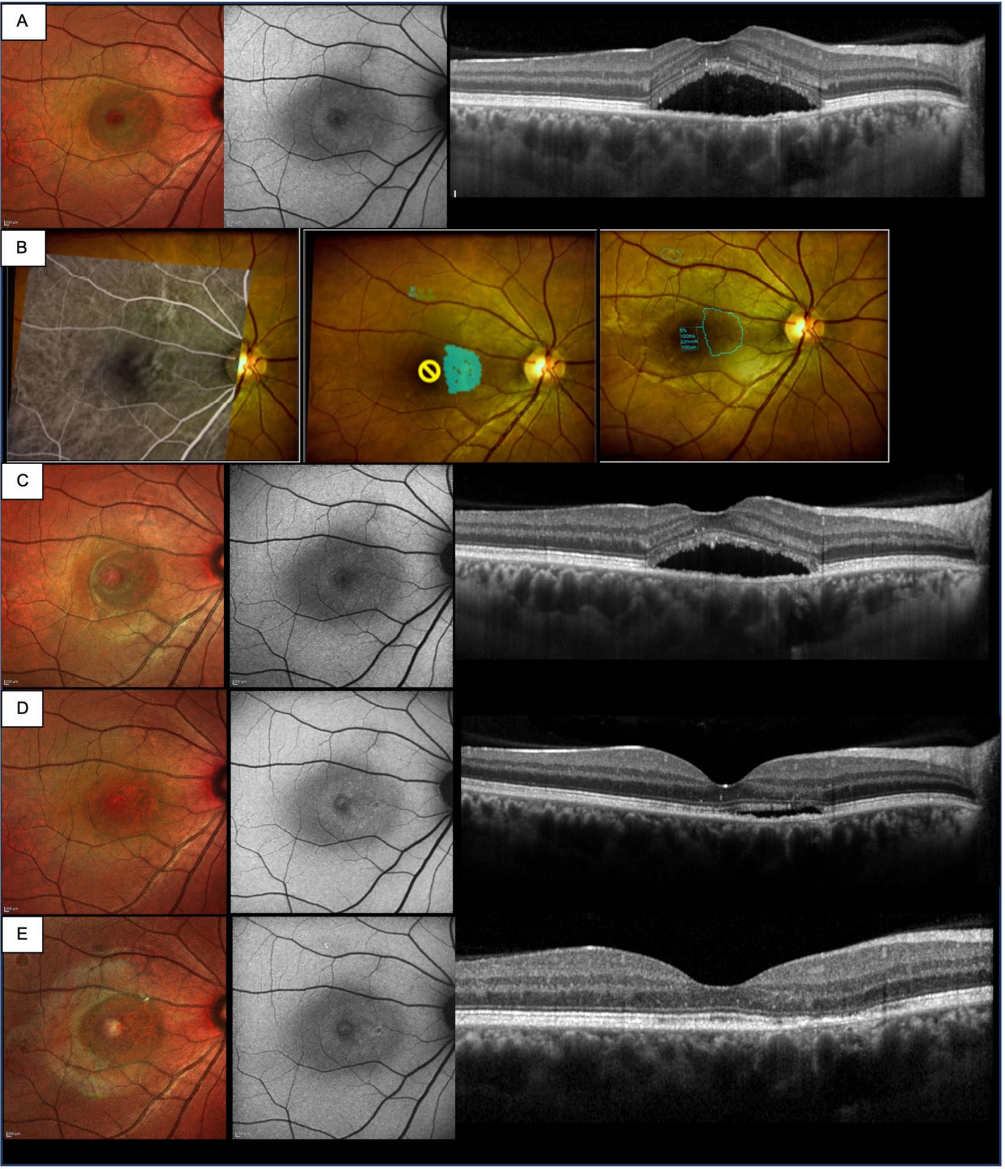
40 years-old males with recurrent cCSC episode for 6 months previously treated with oral acetazolamide without resolution. (**A**) Baseline MCF, FAF, SD-OCT images. (**B**) Navilas laser subthreshold photocoagulation images (fundus image with superimposed ICGA image, fundus image with superimposed titration and laser planning area, fundus image with superimposed applied laser parameters). (**C**–**E**) Follow up MCF, FAF, SD-OCT images at 30, 60 and 90 days after treatment.

Figure 2 reports multimodal imaging of a 40 years-old male cCSC patient at baseline and during the 3-month follow up showing resolution of foveal subretinal fluid after navigated subthreshold laser treatment.

### Safety

No laser spots were visualized in any post laser visits by biomicroscopy, spectral domain OCT (SD-OCT), or fundus autofluorescence (FAF). None of the patients had any laser procedure-related complications or eplerenone-related complications.

## Discussion

This study compared the short-term efficacy of navigated SML and eplerenone in cCSC during a 3-month follow up study. Both treatments were effective showing recovery of retinal morphology and improvement of visual function.

Mean BCVA improved significantly and both CMT and FSRFT reduced significantly over time (*p* < 0.001) showing greater variation in SML group compared to eplerenone group (*p* < 0.001 and *p* = 0.043).

The complete resolution of foveal SRF was present in SML group in 55.6% and in eplerenone group in 66.7% during a 3-month follow up with a statistically significant difference between the two groups at 60 days (*p* = 0.019).

It can be assumed that SML reduced SRF in all patients in a more evenly and slowly modality, whilst eplerenone acts more rapidly in patients who respond to therapy.

Several studies demonstrated the safety and the efficacy of the subthreshold laser using prevalently a diode source of 810 nm or yellow 577 nm micropulse laser in single or multiple sessions to treat cCSC with percentage of complete resolution ranging between 55 and 79% of patients and at least partial resolution in 75–100%^25–34^.

Luttrull et al. analyzed the effects of SML in a small group of 11 patients with disease duration between 1 and 7 months, finding a total resolution of foveal SRF in all cases regardless of disease duration^29^.

In 2015, Scholz et al., evaluated the efficacy of SML in patients with cCSC resistant to PDT in a study with a mean follow up of 5.0 ± 3.7 months and a number of treatments from 1 to 3 and showed the resolution of SRF in 24% of patients and 75% showed at least a partial resolution of fluid^31^.

Maruko et al. reported a complete resolution in 9 of 14 eyes (64.3%) cCSC patients with a mean duration of symptoms of 2.4 months. The mean time of resolution after SML was of 1.9 months and the average number of treatments was 1.34^33^.

Similar percentages of resolution were found in a recent study by Sun et al. that reported at week 12 a percentage of resolution of 63.63% after a single session of SML treatment in CSC patients with foveal SRF involving the center of the macula within 6 months. In this study SRF had been totally resolved in about 50% patients at week 3 and 65% at week 12^34^.

Similar percentages of SRF resolution in cCSC were reported after eplerenone treatment ranging from 13 to 31% at about 2 months and from 29 to 100% during a 4-month follow up^35–37^.

The first use of Eplerenone for cCSC was reported in a non-randomized pilot study showing total reabsorption of SRF in 81.8% of patients eyes during a 3-month follow up^38^.

### Toto et al. reported resolution of SRF in 71.4% of eyes within 60 days and 100% at 120 days^39^

Previous comparative studies were also conducted to evaluate the efficacy of SML compared to PDT, anti vascular endothelial growth factor (VEGF) treatment or eplerenone^18,40–47^.

Vignesh et al. compared the outcomes of SML and eplerenone treatments in cCSC eyes during a mean follow up of 8 months and reported comparable visual outcomes between the two treatments with 42.8% and 20% of complete resolution of SRF in SML group and eplerenone group respectively^18^.

Differently from our clinical study that used a navigated SML device in this retrospective comparative study Vignesh focused on traditional SML system. The different percentage of resolution was probably related to different setting parameters of SML protocol treatment (power of 50% of titration energy; 200 ms duration). In addition, a different status of the retina and RPE at baseline mainly due to the duration of the disease could account for the lower resolution rate of SRF in Vignesh study compared to our results.

The effect of different treatments for CSC in the several clinical studies varies according to different CSC-related ocular features and laser parameters^18,25–47^.

Chronic disease causes permanent retinal damage and the longer the disease duration and the less likely is the potential for treatment response and visual recovery.

Patients treated earlier recover good visual acuity and achieve restored retinal morphology. In contrast, several reports showed limited satisfactory functional results in long lasting chronic disease.

Scholz et al., in a meta-analysis including the results of 12 studies evaluating efficacy of SML in cCSC found an average improvement in BCVA of 6.34 Early Treatment Diabetic Retinopathy Study (ETDRS) letters in patients with a disease duration varying from ≥ 4 weeks to ≥ 6 months^15^. In one of the reviewed studies conducted by Scholz et al.

Mean duration of the disease before SML was 3.9 years (± 4.2, range 1.7 month–19 years) with a significant increase of BCVA from 0.39 ± 0.24 to 0.31 ± 0.27 logMAR after therapy^31^. In this study patients with a disease duration < 1 year showed a better treatment response after SML but not after PDT compared with patients with a disease duration of > 1 year^15^.

Presence of widespread RPE atrophy in cCSC patients treated with eplerenone showed lower gains of visual acuity compared to patients with more preserved RPE status^36^. Cakir et al. included in their study 24 patients with a mean disease duration of 326 days (120–3032), 6 of these patients presented with widespread RPE atrophy at baseline and showed a loss in BCVA from a mean of 0.47 at baseline to 0.52 logMAR at the last follow-up compared to patients with nonresolving CSC and subtle RPE changes that improved from 0.31 at baseline to 0.24 at the last follow-up treatment. The presence of an intact RPE layer and integrity of the ellipsoid zone at baseline were associated with a tendency towards a favourable visual outcome was associated with an improvement in BCVA.

In our study the good functional outcome with mean BCVA at 3 months of 0.1 logMAR in SML group and 0.2 logMAR in eplerenone group is probably due to the short disease duration (within 8 months) with most patients showing at baseline limited retinal damage. In our cohort 12 out of 18 patients of the SML group and 10 out of 18 patients of the eplerenone group showed subtle RPE changes or focal atrophy without foveal involvement.

Similar visual outcomes were reported by previous studies of SML in CSC patients with limited disease duration with post treatment BCVA included between 0.2 to 0.0 logMAR^29,32–34^.

The favourable morphological outcome also detected in our series with 55.6% of patients showing complete SRF resolution after a single SML treatment at 3 months was in agreement with the results of other studies reporting SRF resolution percentages included between 24 and 64% in CSC with a mean follow up of 5 months duration or less^31,34^.

In the eplerenone group the percentage of resolution of SRF of 66.7% at 60 and 90 days was slightly higher than the percentages reported in the literature ranging from 13 to 57.1% at about 2 months and from 29 to 61% at about 4 months after treatment respectively^35–37,39^.

The significant statistical difference concerning proportion of patients with SRF resolution at 60 days between the two treatments cannot be related to the status of the neurosensory retina and RPE associated to the duration of the disease considering the similar retinal condition between the two groups.

It can be assumed that SML mechanism based on normalization of RPE function and consequent reabsorption of SRF is slower compared to eplerenone mechanism of action.

The anatomical and functional outcome after SML and eplerenone treatment is likely due the good retinal status at baseline related to the relative short disease duration of our patients.

When CSC becomes a chronic disease multifocal or diffuse RPE disruption and atrophy can occur throughout the posterior pole. The condition of RPE is very important in the pathophysiology and prognosis of the disease. Chronic atrophic CSC and widespread RPE atrophy at baseline have been associated with a slower therapy response and lower visual gains compared to patients with subtle RPE changes. Short disease duration has been associated with more favourable outcomes both for SML and eplerenone treatments^34,36,48^.

In addition, compared to traditional SML laser the navigated laser treatment with fundus fluorescein angiography (FFA)/indocyanine green angiography (ICGA) images overlaid on the fundus image allowed to precisely treat leakage areas perfectly targeting the diseased RPE.

### The main limitation of this study is the limited sample size of patients and the similar baseline characteristics of the patients included in the study

Larger sample of patients with more varied disease features at baseline such as a wider range of disease duration will help to understand treatment efficacy in different status of damaged retina.

In conclusion, our study demonstrates that patients with cCSC may benefit from both SML and eplerenone treatments with more favourable outcomes in terms of CMT reduction with short term SML treatment and more rapid resolution of SRF in the eplerenone group.

## Materials and methods

### Study participants

A total of 36 eyes of 36 patients suffering from cCSC and monolateral FSRF treated with navigated SML (Navilas® 577s; OD-OS GmbH, near Berlin, Germany) (18 eyes, SML group) and oral eplerenone (18 eyes, eplerenone group) were enrolled in this retrospective observational study. Patients referred to the retina center of the Ophthalmology Clinic of University “G. d’Annunzio”, Chieti-Pescara, Italy between January 2018 and February 2021. The study adhered to the tenets of the Declaration of Helsinki and our Institutional Review Board (Department of Medicine and Science of Ageing, University G. D'Annunzio Chieti-Pescara), approved the retrospective chart review. An informed consent was obtained from all patients.

Inclusion criteria were: (1) patients aged between 18 and 60 years; (2) disease duration equal or more than 6 months as first episode or recurrence; (3) presence of foveal SRF on SD-OCT. Exclusion criteria were as follows: (1) previous treatments such as PDT, focal photocoagulation and intravitreal injections of anti- VEGF; (2) use of steroid systemically; (3) other retinal diseases; (4) pregnancy.

### Examinations

All patients received comprehensive ophthalmic examination including BCVA evaluation using Early Treatment Diabetic Retinopathy Study (ETDRS) chart after refraction measurements, Goldmann applanation tonometry, slit-lamp biomicroscopy and indirect fundus ophthalmoscopy. In addition, multicolor imaging (MCI), FFA, and ICGA, FAF, and SD-OCT were performed using Spectralis ® HRA+OCT (Heidelberg Engineering; Heidelberg, Germany).

Best corrected visual acuity, anterior segment biomicroscopy, intraocular pressure (IOP), indirect fundus exam, MCI, FAF and SD OCT were performed at baseline, and then monthly after SML or oral eplerenone as per routine clinical practice. FFA and ICGA were performed at baseline.

### SD OCT analysis

The acquisition protocol for SD OCT included a 49 horizontal raster dense linear B-scans centered on the fovea. A horizontal and vertical B-scans centered on the fovea with enhanced depth imaging (EDI) mode were acquired in all patients.

All acquisitions following baseline visit were acquired using the follow-up function.

Central macular thickness was measured using the central 1-mm-diameter circle of the ETDRS thickness map.

Foveal subretinal fluid thickness defined as the vertical distance between the end of the outer segment and the RPE at the foveal center was measured using the inbuilt manual caliper.

Subfoveal choroidal thickness measured vertically from the outer border of the RPE to the inner border of the sclera was measured using the inbuilt manual caliper on EDI OCT scans.

#### Multimodal imaging assessment of RPE atrophy

The RPE changes observed at MCI, FAF and SD-OCT were analyzed at baseline. They were categorized as widespread RPE atrophy, and focal RPE atrophy.

Focal RPE atrophy was defined as a single RPE atrophy area in the posterior pole characterized by a hypopigmentation with possible increased visibility of choroidal vessels at MCI, hypoautofluorescence on FAF combined with attenuation or disruption of RPE band with possible overlying photoreceptor degeneration on SD-OCT.

Widespread atrophy was defined as multifocal (> 1) RPE atrophy areas.

### Choroidal hyperpermeability assessment

Choroidal hyperpermeability was evaluated in the late phase of ICGA and as previously described was as multifocal areas of hyperfluorescence with blurred margins within the choroid^49^.

### 577-nm micropulse laser treatment

The Navilas® Laser System 577s Prime, a 577-nm yellow laser system for navigated focal and peripheral laser treatments (OD-OS GmbH, Warthestr. 21 14513 Teltow Germany), was used. The micropulse treatment parameters were standardized for all patients, with 100 μm spot size and 100 ms duration with 5% duty cycle. The power was individualized in every patient after energy titration before treatment in a normal area of retina outside the vascular arcade. The titration was performed in microsecond mode with 5% duty cycle starting from 700 mW power with single spots with 50 mW increasing power until the appearance of a barely visible burn on the retina, this was used as the threshold limit. The final laser treatment power was set at 30% of titrated energy and ranged from 210 to 260 mW. The micropulse laser in a multiple dense spot pattern was delivered to the leakage areas on a mid-phase FFA or hypercianescent areas on midphase ICGA images that were imported to the laser device, superimposed and aligned with the live image by means of an eye tracking system.

In case of SRF persistence at 90 days control visit there was an indication to a second SML treatment.

### Eplerenone treatment

Oral Eplerenone at a daily dose of 25 mg for the first week and then 50 mg planned for the following weeks until SRF resolution and for not more than 6 consecutive months.

### Management of unsuccessful patients

In case of SRF persistence at 6 months patients treated with SML or oral eplerenone were shifted to other treatments or combined treatments could be proposed.

### Outcome measures

The primary outcome measures were BCVA, CMT, FSRFT and SFCT that were collected at baseline and at each follow-up visit up to 3 months.

### Statistical analysis

Jarque–Bera test showed a normal distribution of continues variables. Descriptive statistics included frequencies and proportions for categorical variables, mean and standard deviation for continuous. Differences between the two groups were tested by the Student t test and Pearson’s chi-square test for continuous and categorical variables, respectively. Planned contrasts’ on the t-statistic were used to determine mean differences between and within groups. A mixed model ANOVA was applied to compare SML and oral eplerenone groups (between subject effect) and time points (within subject effect).

For treated groups we analyzed all time-to-event distributions using the Kaplan–Meier’s method. Kaplan–Meier curve illustrated probability to be retreated over a time of 90 days. All statistical tests were 2-sided, with a significance level set at *p* < 0.05. Analyses were performed using the R software environment for statistical computing and graphics (version 3.4.1; http://www.r-project.org/).
